# Assessment of calcium intake by adolescents

**DOI:** 10.1590/0103-0582201432211913

**Published:** 2014-06

**Authors:** Cristiane Franco de Oliveira, Carla Rosane da Silveira, Mariur Beghetto, Paula Daniel de Mello, Elza Daniel de Mello

**Affiliations:** 1 UFRGS, Porto Alegre, RS, Brasil

**Keywords:** calcium, dietary, diet, adolescent, osteoporosis

## Abstract

**OBJECTIVE:**

To evaluate the daily calcium intake of adolescents in schools from Chapecó, Santa Catarina, Southern Brazil, to check if calcium intake is in accordance with the Dietary Reference Intakes (DRI), and to investigate variables associated with daily calcium intake.

**METHODS:**

Cross-sectional study approved by the Institutional Review Board and developed in 2010. Students of the 8^th^ grade completed questionnaires with personal data and questions about the calcium-rich foods intake frequency. In order to compare students with adequate (1300mg) or inadequate intake of calcium/day (<1300mg), parametric and nonparametric tests were used.

**RESULTS:**

A total of 214 students with a mean age of 14.3±1.0 years were enrolled. The median daily calcium intake was 540mg (interquartile range - IQ: 312-829mg) and only 25 students (11.7%) had calcium intake within the recommendations of the DRI for age. Soft drink consumption ≥3 times/week was associated with a lower intake of calcium.

**CONCLUSIONS:**

Few students ingested adequate levels of calcium for the age group. It is necessary to develop a program to encourage a greater intake of calcium-rich foods in adolescence.

## Abstract (es)

**OBJETIVOS:**

Evaluar la ingestión diaria de calcio de los adolescentes de escuelas del municipio de Chapecó, Santa Catarina, verificar si esa ingestión de calcio está en conformidad con las *Dietary Reference Intakes* (DRI) e investigar los factores que pueden interferir en la ingestión diaria de calcio.

**MÉTODOS:**

Estudio transversal, aprobado por el Comité de Ética e Investigación, desarrollado en 2010. Alumnos del octavo año contestaron a cuestionarios con datos personales y preguntas sobre la frecuencia de la ingestión de alimentos ricos en calcio. Se presentaron características de la población en promedio y desviación estándar o mediana (mínimo y máximo). Para comparar escolares con ingestión adecuada (1300mg) o inadecuada de calcio/día (<1300mg), se utilizaron pruebas paramétrica y no paramétrica.

**RESULTADOS:**

Se evaluaron 214 alumnos con promedio de edad de 14,3±1,0 años. La mediana de consumo diario de calcio fue de 540mg (intervalo intercuartil - IQ: 312-829mg) y solamente 25 alumnos (11,7%) presentaron ingestión de calcio dentro de las recomendaciones de las DRI para la edad. El consumo de refrescos ≥3 veces/semana se mostró asociado a menor consumo de calcio.

**CONCLUSIONES:**

Pocos alumnos ingirieron calcio en niveles adecuados para la franja de edad, lo que hace necesario desarrollar un programa de incentivo a la mayor ingestión de alimentos ricos en calcio.

## Introduction

It is estimated that the percentage of chronic noncommunicable degenerative diseases, such as osteoporosis, will increase by 57% by 2020. Osteoporosis is defined by the World Health Organization (WHO) as a systemic metabolic disease, characterized by reductions in bone mass and deterioration of the microarchitecture of bone tissue. Osteopenia, in turn, is a reduction in bone mass without compromise to microarchitecture. Despite these different definitions, the consequence of both is increased bone fragility and, therefore, greater susceptibility to fractures^(^ 1 ^)^.

Bone mineral density (BMD) in adulthood is dependent on the peak bone mass acquired by the end of the second decade of life. Although there is no consensus on the age at which peak bone mass is reached, several authors believe that around 40% of bone mass is accumulated by 11-14 years of age in girls and 13-17 years of age in boys^(^ 1 ^,^ 2 ^)^. Good bone structure is of fundamental importance and is considered one of the most effective means of preventing osteoporosis at advanced ages^(^ 1 ^)^.

The principal determinant of bone formation is calcium in the diet. If dietary availability of calcium is inadequate, the body will transport calcium from the bones into the bloodstream, increasing their fragility^(^ 3 ^)^. Calcium requirements vary by age group and are higher during periods of rapid growth, such as during adolescence, when the requirement is around 1300mg/day^(^ 2 ^)^.

An adequate intake of calcium during childhood and adolescence is therefore fundamental for prevention of osteoporosis, which is the reason for evaluating the calcium intake profiles of adolescents. Several studies conducted at other centers have shown that the younger population does not meet the daily recommendations for age and sex^(^ 4 ^-^ 9 ^)^. While the city of Chapecó, SC, has some of the best socioeconomic indicators in Brazil, the majority of children enrolled in primary education are studying in public schools, which are covered by a school meals system. Accumulation of data on the calcium intake in the city could provide a basis for a realignment of the school meals menus, in addition to promoting educational interventions in the city. Therefore, the objective of this study was to compare mean calcium intakes among adolescents at schools in Chapecó with those recommended by the Dietary Reference Intakes (DRI) and evaluate factors possibly associated with intakes.

## Method

This project was approved by the Research Ethics Committee at the Hospital de Clínicas de Porto Alegre (protocol number 10-0214). It is a cross-sectional study of eighth-grade students at public (state and municipal) and private schools in the municipal district of Chapecó during the 2010 academic year. The sample frame took account of the proportions of numbers of students enrolled in the public and private systems. Clusters (classes) were chosen from schools and all students in each cluster were considered potentially eligible. Adolescents were excluded if they had chronic diseases (defined as those needing continuous treatment) or mental and psychiatric disorders that could interfere with understanding or participation, if they reported taking calcium as a drug treatment, were absent from school on the day of data collection or if they were enrolled at indigenous schools.

Students completed a questionnaire comprising objective questions on their socioeconomic characteristics, dietary habits and physical activity practices and a food frequency questionnaire^(^ 10 ^)^ that covered foods rich in calcium and was tailored to the dietary habits of the adolescents in the region. The list of food items was based on a previously validated food frequency questionnaire^(^ 10 ^,^ 11 ^)^.

Before administration of the questionnaires, which took place in a classroom during lesson time, and for all classes selected, the lead researcher explained the answer method and the meaning of the questions, remaining in the classroom while the students completed the questionnaires. The portion sizes for each food were illustrated on a poster with color photographs. The portion sizes were defined as recommended by Monteiro^(^ 12 ^)^.

The food frequency questionnaire for calcium asked about the quantities (in portions) and frequency of intake of the following foods: milk, yoghurt, ricotta, cheeses (*minas*, *lanche *and *mussarela*), processed cheese spread, cheese sourdough, oats, beans, dark green vegetables (water cress, rocket, collard greens, spinach and broccoli), cauliflower, fish (*pescada*), cake, ice cream and sweets containing milk (crème caramel). Foods were classified by frequency of consumption into five categories: never eaten or rarely eaten in the last year (N) or the number of times, from one to ten, and a letter to indicate the period, per day (D), per week (W), per month (M) or per year (Y). Portion sizes were classified into four categories: "S" - portion smaller than the average portion as shown on the poster; "M" - equivalent to the average portion; "L" - larger than the average portion; "VL"- much larger than the average portion^(^ 12 ^)^.

A descriptive analysis was conducted of the general characteristics of the students and strata by education system: private schools, state schools or municipal schools. Both parametric and nonparametric tests were employed to compare groups of schoolchildren whose dietary calcium intake was adequate according to their DRIs with those whose intake was inadequate. Continuous variables with symmetrical distribution were expressed as means and standard deviations; other variables were expressed as medians with interquartile ranges. The significance level was set at 5% and analyses were conducted using the Statistical Package for the Social Sciences (SPSS) 18.

The sample size calculation was based on the number of students enrolled in the eighth grade in Chapecó (n=3,054) and their distribution across private schools (n=220; 7.2%), the municipal education system (n=797; 26.1%) and the state education system (n=2,037; 66.7%). Based on the findings of a study conducted in Osasco, SP, Brazil^(^ 5 ^)^, which found that from 2.8 to 6.2% had adequate intakes, and accepting a sampling error of 5%, the sample size was estimated at 44 students. In view of the fact that cluster sampling had been adopted, one class was selected from a private school, two from municipal schools and six from schools run by the state education authority, in order to respect the proportionality of distribution of students and classes across private, municipal and state schools.

## Results

A total of 214 adolescents were investigated, predominantly from public schools (95%), with distribution in social classes A (9.1%), B (61.6%) and C (29.3%). Nine percent of the schoolchildren were only children, 34.8% had one sibling, 24.8% had two siblings and the remainder (39.4%) had three or more siblings. The remaining characteristics of the study participants are shown in Table 1.

**Table 1:**
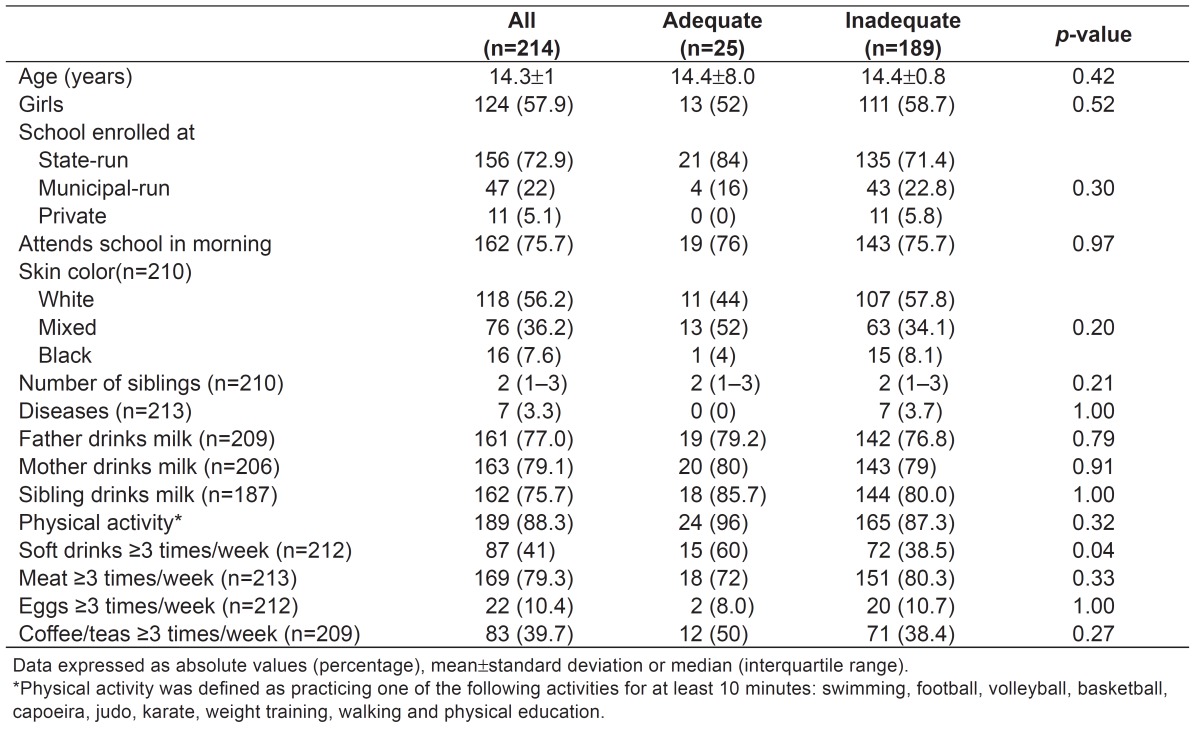
General characteristics of adolescents, by adequate/inadequate calcium intake according to Dietary Reference Intakes

Median daily calcium intake per student was 540mg (interquartile range - IQ: 312-829). Just 25 schoolchildren (11.7%), all at public schools, had calcium intake within the daily recommendations for their age group.

Of the 207 schoolchildren who provided information about breakfast, 49.3% reported having breakfast every day, while 20% stated they almost never had breakfast and 30.7% that they never had breakfast.

Regular intakes of foods that can be associated with calcium absorption, such as soft drinks, meat, eggs and tea or coffee, except milk and its derivatives, are shown in Table 1. 

Univariate analysis did not detect a significant difference between percentage of students at public and private schools with calcium intakes in line with the DRI recommendations (12.3 versus 0%; *p*=0.37).

An association was observed between drinking soft drinks three or more times/week and less adequate calcium intake (*p*=0.04). No other significant differences were observed between schoolchildren with and without adequate calcium intake (Table 1).

## Discussion

This study shows that the eighth-grade adolescents from the city of Chapecó have calcium intakes below what is recommended by the DRIs for their age group.

This study is representative of the population of eighth-grade schoolchildren in the municipal district of Capac, who proved similar to populations described in other studies conducted in Brazil and elsewhere. The most similar study in terms of the age group investigated was conducted in the city of Osasco, also in Brazil^(^ 5 ^)^. Comparison with the study in Osasco reveals that a larger proportion of the students assessed in Chapecó have adequate calcium intakes for their age group (11.7%), since in the Osasco study just 6.2% of males and 2.8% of females had intakes of 1200mg/day or more, which is the minimum level for the age group in the study. In contrast, a study by Rodrigues et al, that investigated mean daily calcium intake among catwalk models found that 18.2% of those adolescents had adequate intake^(^ 6 ^)^. Peters et al investigated calcium and vitamin D intake in postpubescent adolescents, observing that just 3.8% of them consumed the recommended quantity (682.2±132.2mg/day)^(^ 7 ^)^. Santos et al reported mean calcium consumption by adolescents of 703.7±396.0mg/day^(^ 9 ^)^.

Assessing the mean quantity of calcium consumed by the adolescents from Chapecó, it is observed that the mean calcium intake of 540mg/day was lower than reported in studies by Lerner et al, in Osasco (600mg/day)^(^ 5 ^)^, Rodrigues et al, with adolescent catwalk models (700mg/day)^(^ 6 ^)^, Novotny et al, whose sample was of Asian and Hispanic adolescents (998mg/day)^(^ 4 ^)^, Peters et al, who assessed postpubescent adolescents and young adults (682mg/day)^(^ 8 ^)^, and also than Santos et al, who analyzed mean calcium intake in adolescents (703.7mg/day)^(^ 9 ^)^.

Another important point is that students who reported drinking cola-style soft drinks three or more times per week had lower daily calcium intake (*p*=0.04). On the basis of this finding, it could be concluded that, in addition to consuming fewer foods rich in calcium, such as milk and dairy products, these students may also have calcium absorption compromised by drinking more soft drinks. The results of the present study did not reveal any associations between consumption of other foods that interfere with calcium absorption (meat and eggs - sources of proteins - and tea/coffee - sources of caffeine) and consumption of foods that are sources of calcium.

This study also failed to detect any significant association between greater intake of milk by family members and greater consumption by the student, although the data did show that students' siblings consumed more than their parents.

There was a considerable, although not significant difference in terms of physical activity between students whose calcium intake was adequate according to the Food and Agriculture Organization (FAO)/WHO^(^ 13 ^)^ and those whose intakes were not. This is extremely important, since students whose calcium intakes were adequate were also benefiting from better absorption of the mineral, since physical activity facilitates absorption^(^ 14 ^)^.

There was no evidence of differences in calcium intake between the sexes. Boys and girls were equal in terms of the quantities of calcium consumed, in common with studies undertaken in Osasco^(^ 5 ^)^ and Ouro Preto, MG, both in Brazil^(^ 9 ^)^. There was also no difference in calcium intake between students who studied in the mornings and those who studied in the afternoons.

Osteopenia and osteoporosis are common problems in adulthood, but they can be prevented during adolescence through healthy dietary habits. In addition to improving quality of life for individual people, this simple practice would reduce public spending on healthcare^(^ 15 ^-^ 18 ^)^. It is therefore necessary that health professionals exert themselves to stimulate increased consumption of food rich in calcium by adolescents, in order to prevent osteoporosis and its consequences. In Chapecó, after the results of this research were returned to the schools, their menus were modified to increase the offer of milk-based products in an attempt to meet the daily calcium requirements of the age group studied.

It was concluded that the majority of this sample was not consuming adequate levels of calcium for their age group and sex, according to the FAO/WHO recommendation of 1300mg/day^(^ 1 ^,^ 13 ^)^.

The limitations of this study are related to the applicability of the questionnaire, since it was dependent on complete and honest responses from the adolescents. In common with other studies that have been mentioned, this investigation found low calcium intake in the adolescent population of the city of Chapecó, which is a public health problem that is not restricted to this locale.

The findings of this study show that it is necessary to develop a program to encourage greater consumption of calcium in the age group investigated. Providing adolescents and their families/carers with information and practical examples of sources of foods rich in calcium, their quantities in each food and the factors that facilitate or interfere with absorption would improve understanding of the importance of formation and maintenance of bone mineral density for prevention of osteopenia and osteoporosis.

## References

[1] Organizacíon Mundial de la Salud (2013). Dieta, nutricíon y prevencíon de enfermedades crônicas.

[2] Silva CC, Teixeira AS, Goldberg TB (2004). The impact of calcium ingestion on the bone
mineralization in adolescents. Rev Nutr.

[3] Grüdtner VS, Weingrill P, Fernandes AL (1997). Absorption aspects of calcium and vitamin D
metabolism. Rev Bras Reumatol.

[4] Novotny R, Boushey C, Bock MA, Peck L, Auld G, Bruhn CM (2003). Calcium intake of Asian, Hispanic and white
youth. J Am Coll Nutr.

[5] Lerner BR, Lei DL, Chaves SP, Freire RD (2000). Consumption of calcium by adolescents from public school
em Osasco, São Paulo, Brazil. Rev Nutr.

[6] Rodrigues AM, Cintra IP, Santos LC, Martini LA, Mello MT, Fisberg M (2009). Bone mineral density, body composition, and food intake
of adolescent runway models. J Pediatr (Rio J).

[7] Peters BS, dos Santos LC, Fisberg M, Wood RJ, Martini LA (2009). Prevalence of vitamin D insuficiency in Brazilian
adolescents. Ann Nutr Metab.

[8] Peters BS, Verly E, Marchioni DM, Fisberg M, Martini LA (2012). The influence of breakfast and dairy products on dietary
calcium and vitamin D intake in postpubertal adolescents and young
adults. J Hum Nutr Diet.

[9] Santos LC, Martini LA, Freitas SN, Cintra IP (2007). Calcium intake and anthropometric indicators in
adolescents. Rev Nutr.

[10] Uenishi K, Ishida H, Nakamura K (2008). Development of a simple food frequency questionnaire to
estimate intakes of calcium and other nutrients for the prevention and management
of osteoporosis. J Nutr Sci Vitaminol (Tokyo).

[11] Ataíde e Silva  T, Vasconcelos SM (2012). Methodological procedures used in food frequency
questionnaires made in Brazil: a systematic review. Rev Nutr.

[12] Monteiro JP (2007). Nutrição e metabolismo - consumo alimentar: visualizando
porções.

[13] World Health Organization (2013). Vitamin and mineral requirements in human nutrition.

[14] Almeida BR, Rodrigues RL (1997). Influência da atividade física e da ingestão de cálcio
na osteoporose. Motriz.

[15] Pereira GA, Genaro PS, Pinheiro MM, Szejnfeld VL, Martini LA (2009). Cálcio dietético - estratégias para otimizar o
consumo. Rev Bras Reumatol.

[16] Kowalski SC, Sjenzfeld VL, Ferraz MB (2001). Utilização de recursos e custos em
osteoporose. Rev Assoc Med Bras.

[17] Lau EM (2001). Epidemiology of osteoporosis. Best Pract Res Clin Rheumatol.

[18] Pinheiro MM, Schuch NJ, Genaro PS, Ciconelli RM, Ferraz MB, Martini LA (2009). Nutrient intakes related to osteoporotic fractures in
men and women - The Brazilian Osteoporosis Study (BRAZOS). Nutr J.

