# Supplementary material for: Assessment of calcium intake by adolescents
Source: Rev Paul Pediatr. 2014 Jun;32(2):216–20. doi: 10.1590/0103-0582201432211913 (PMC4183011; doi:10.1590/0103-0582201432211913)
Supplement: Translation

# Translation: Avaliação do consumo de cálcio por adolescentes

**Authors:** Cristiane Franco de Oliveira, Carla Rosane da Silveira, Mariur Beghetto, Paula Daniel de Mello, Elza Daniel de Mello

**Affiliations:** 1 UFRGS, Porto Alegre, RS, Brasil

**Keywords:** cálcio na dieta, dieta, adolescente, osteoporose

## Abstract

**OBJETIVO:**

Avaliar a ingestão diária de cálcio dos adolescentes de escolas do município de Chapecó, SC, verificar se essa ingestão de cálcio está de acordo com as *Dietary Reference Intakes* (DRI) e investigar os fatores que podem interferir na ingestão diária de cálcio.

**MÉTODOS:**

Estudo transversal, aprovado pelo Comitê de Ética e Pesquisa e desenvolvido em 2010. Alunos de oitava série responderam questionários com dados pessoais e perguntas sobre a frequência da ingestão de alimentos ricos em cálcio. Para comparar escolares com ingestão adequada (1300mg) ou inadequada de cálcio/dia (<1300mg), utilizaram-se testes paramétrico e não paramétrico.

**RESULTADOS:**

Avaliaram-se 214 alunos com média de idade de 14,3±1,0 anos. A mediana de consumo diário de cálcio foi de 540mg (intervalo interquartil - IQ: 312-829mg) e somente 25 alunos (11,7%) apresentaram ingestão de cálcio dentro das recomendações das DRI para a idade. O consumo de refrigerante ≥3 vezes/semana mostrou-se associado a menor consumo de cálcio.

**CONCLUSÕES:**

Poucos alunos ingeriam cálcio em níveis adequados para a faixa etária, o que torna necessário desenvolver um programa de incentivo à maior ingestão de alimentos ricos em cálcio.

## Introdução

Estima-se que a porcentagem de doenças crônico-degenerativas não transmissíveis, como a osteoporose, aumente em 57% até o ano de 2020. A osteoporose é definida pela Organização Mundial da Saúde (OMS) como uma doença metabólica sistêmica, caracterizada pela diminuição da massa óssea e pela deterioração da microarquitetura do tecido ósseo. A osteopenia, por sua vez, é a diminuição da massa óssea sem o comprometimento da microarquitetura. Mesmo com definições distintas, ambas têm como consequência o aumento da fragilidade óssea e, portanto, uma maior suscetibilidade a fraturas^(^ ^1^ ^)^.

A densidade mineral óssea (DMO) dos adultos depende do pico de massa óssea adquirido até o final da segunda década de vida. Embora não haja consenso quanto à idade na qual ocorre o pico de massa óssea, vários autores consideram que cerca de 40% da massa óssea é acumulada entre 11 e 14 anos nas meninas e entre 13 e 17 anos nos meninos^(^ ^1^ ^,^ ^2^ ^)^. Uma boa formação óssea é fundamental, sendo considerada um dos meios mais eficazes para prevenir a osteoporose em idades avançadas^(^ ^1^ ^)^.

O principal determinante da formação óssea é o cálcio presente na dieta. Caso não haja uma disponibilidade dietética adequada de cálcio, o organismo mobilizará o cálcio presente nos ossos para a corrente sanguínea, aumentando, assim, a sua fragilidade^(^ ^3^ ^)^. A necessidade de cálcio varia conforme a faixa etária, sendo maior em períodos de crescimento rápido, como na adolescência, ao redor de 1300mg/dia^(^ ^2^ ^)^.

Dessa forma, a ingestão adequada de cálcio na infância e na adolescência é fundamental para prevenir a osteoporose, justificando-se a avaliação do perfil de ingestão de cálcio dos adolescentes. Diferentes estudos, em outros centros, demonstraram que a população infantojuvenil não atinge às recomendações diárias para sexo e idade^(^ ^4^ ^-^ ^9^ ^)^.

A despeito de a cidade de Chapecó, SC, apresentar indicadores socioeconômicos entre os mais elevados do país, a maior parte das matrículas no ensino fundamental está alocada em escolas públicas, sob cobertura de merenda escolar. Conhecer os dados relativos ao consumo de cálcio nessa cidade poderá subsidiar o redirecionamento do cardápio da merenda, além de promover ações educativas nesta cidade. Nesse sentido, o presente estudo teve como objetivo comparar a ingestão média de cálcio em adolescentes de escolas de Chapecó com o recomendado pela *Dietary Reference Intakes* (DRI) e avaliar possíveis fatores associados à ingestão.

## Método

O projeto foi aprovado pelo Comitê de Ética em Pesquisa do Hospital de Clínicas de Porto Alegre. Trata-se de um estudo transversal, que incluiu estudantes das oitavas séries de escolas públicas (estaduais e municipais) e privadas do município de Chapecó no ano letivo de 2010. A amostra respeitou a proporcionalidade do número de alunos matriculados na rede pública e privada. Sortearam-se conglomerados (turmas) das escolas e consideraram-se todos os alunos do conglomerado como potencialmente elegíveis. Não se incluíram portadores de doenças crônicas (definidas como aquelas que necessitam de terapia de uso contínuo) ou mentais e psiquiátricas que pudessem interferir na compreensão ou participação, além de alunos que relataram ingerir cálcio como tratamento medicamentoso, os ausentes no dia da coleta dos dados e os matriculados em escolas indígenas. 

 Os alunos responderam a um questionário composto de questões objetivas referentes às suas características socioeconômicas, hábitos alimentares e prática de atividade física e a um questionário de frequência alimentar^(^ ^10^ ^)^, que englobava alimentos ricos em cálcio, respeitando os hábitos alimentares dos adolescentes da região de estudo. Elaborou-se a relação dos itens alimentares por meio de um questionário de frequência alimentar previamente validado^(^ ^10^ ^,^ ^11^ ^)^.

 Antes da aplicação dos questionários, realizada em sala de aula no horário letivo, em todas as turmas selecionadas, a pesquisadora principal esclareceu o modo de preenchimento e o significado das questões, mantendo-se presente enquanto os alunos o preenchiam. O tamanho das porções para cada alimento foi exemplificado por meio de um pôster com fotos coloridas. A definição dos tamanhos das porções seguiu a recomendação de Monteiro^(^ ^12^ ^)^. 

O questionário de frequência alimentar de cálcio investigou a quantidade (em porções) e a frequência de consumo dos seguintes alimentos: leite, iogurte, ricota, queijos tipo minas, lanche e mussarela, requeijão, pão de queijo, aveia, feijão, vegetais verde-escuros (agrião, rúcula, couve, espinafre e brócolis), couve-flor, peixe (pescada), bolo, sorvete e doces com leite (pudim). Classificaram-se os alimentos conforme a frequência de consumo em cinco categorias: nunca comeu ou raramente comeu no último ano (N) ou o número correspondente de vezes - de uma a dez vezes e se por dia (D), por semana (S), por mês (M) ou por ano (A). Para o tamanho da porção, adotaram-se quatro categorias: "P" - porção menor que a porção média apresentada no pôster; "M" - equivalente à porção média; "G" - maior que a porção média; "E"- muito maior que a porção média^(^ ^12^ ^)^.

Realizou-se a análise descritiva das características gerais dos alunos e dos estratos por tipo de matrícula: escolas privadas, estaduais ou municipais. Adotaram-se testes paramétricos e não paramétricos para comparar grupos de escolares cujo consumo de cálcio na dieta estava adequado às DRI's com aqueles sem consumo adequado. Apresentaram-se as variáveis contínuas com distribuição simétrica por meio de média e desvio padrão; as demais, por meio de mediana e do intervalo interquartil. O nível de significância adotado foi de 5% e as análises foram realizadas no *software Statistical Package for the Social Sciences* (SPSS) 18.

Para o cálculo amostral, considerou-se o número de alunos matriculados na oitava série do ensino fundamental de Chapecó (n=3.054) e sua distribuição na rede privada (n=220; 7,2%), na rede municipal (n=797; 26,1%) e na rede estadual (n=2.037; 66,7%). Baseando-se no estudo conduzido em Osasco, SP^(^ ^5^ ^)^, que encontrou adequação de consumo de cálcio de 2,8 a 6,2%, e aceitando-se um erro amostral de 5%, estimou-se a amostra em 44 alunos. Tendo em vista a adoção de amostragem por conglomerados, a fim de se respeitar a proporcionalidade (distribuição) de alunos e turmas na rede privada, municipal e estadual, selecionou-se uma turma da rede privada, duas da rede municipal e seis da rede estadual.

## Resultados

Avaliaram-se 214 adolescentes, predominantemente de escolas públicas (95%), distribuídos nas classes sociais A (9,1%), B (61,6%) e C (29,3%). Enquanto 9% dos escolares eram filhos únicos, 34,8% tinham um irmão, 24,8% tinham dois irmãos e o restante (39,4%) tinha três ou mais irmãos. As demais características dos participantes estão apresentadas na Tabela 1. 

**Tabela 1:** Características gerais entre os grupos de consumo de cálcio adequado e não adequado às Dietary Reference Intakes

A mediana de consumo diário de cálcio por aluno foi de 540mg (intervalo interquartil - IQ: 312-829). Somente 25 escolares (11,7%), todos de escolas públicas, apresentaram consumo de cálcio dentro das recomendações de ingestão diária para a faixa etária. 

Dos 207 escolares que informaram sobre o café da manhã, 49,3% declararam fazer essa refeição diariamente, enquanto outros 20% referiram quase nunca fazê-la e 30,7%, nunca fazê-la. 

O consumo regular de alimentos que podem se associar à absorção do cálcio, tais como refrigerantes, carnes, ovos e chá ou café, excetuando-se leite e derivados, está na Tabela 1.

Em análise univariada, não houve diferença na adequação do consumo de cálcio às recomendações das DRI em escolas públicas e privadas (12,3 *versus* 0%; *p*=0,37). 

Observou-se associação entre consumo de refrigerantes três ou mais vezes/semana e a menor adequação da ingestão de cálcio (*p*=0,04). Não se observaram outras diferenças significativas entre os escolares com e sem adequação no consumo de cálcio (Tabela 1).

## Discussão

O presente estudo demonstrou que os adolescentes de oitava série do município de Chapecó ingerem cálcio em quantidade inferior à recomendada pelas DRI segundo a faixa etária.

O trabalho representa a população de escolares de oitava série do município de Chapecó, a qual se mostrou semelhante à população de outros estudos nacionais e internacionais. O estudo mais próximo quanto à faixa etária avaliada foi o realizado no município de Osasco^(5).^ Ao comparar o presente estudo com o realizado em Osasco, nota-se que um maior número de alunos avaliados em Chapecó ingere quantidades de cálcio adequadas para a faixa etária (11,7%), ao passo que, na pesquisa de Osasco, somente 6,2% dos homens e 2,8% das mulheres apresentaram consumo de 1200mg/dia ou mais, valor mínimo de adequação para a faixa etária neste estudo. Já o trabalho de Rodrigues *et al*, que avaliou a ingestão média de cálcio ao dia de modelos de passarela, encontrou adequação em 18,2% das adolescentes^(^ ^6^ ^)^. Em adolescentes pós-púberes, Peters *et al* avaliaram a ingestão de cálcio e vitamina D e observaram que apenas 3,8% dos indivíduos ingeriam o aporte recomendado (682,2±132,2mg/dia)^(^ ^7^ ^)^. Santos *et al* encontraram ingestão média de cálcio por adolescentes de 703,7±396,0mg/dia^(^ ^9^ ^)^.

Ao avaliar a quantidade média do cálcio ingerido pelos adolescentes de Chapecó, obteve-se a menor ingestão média de cálcio, 540mg/dia, em comparação com os estudos de Lerner *et al*, realizado em Osasco (600mg/dia)^(^ ^5^ ^)^, Rodrigues *et al*, com adolescentes modelos de passarela (700mg/dia)^(^ ^6^ ^)^, Novotny *et al*, cuja amostra era de adolescentes asiáticos e hispânicos (998mg/dia)^(^ ^4^ ^)^, Peters *et al*, que avaliaram adolescentes pós-púberes e adultos jovens (682mg/dia)^(^ ^8^ ^)^, e Santos *et al*, que analisaram a ingestão média de cálcio por adolescentes (703,7 mg/dia)^(^ ^9^ ^)^.

Outro ponto importante é que os alunos que referiram ingerir refrigerante do tipo cola três ou mais vezes na semana apresentaram menor ingestão diária de cálcio (*p*=0,04). Com esse achado, pode-se concluir que, além de ingerirem menos alimentos ricos em cálcio, como leite e derivados, esses alunos, por beberem mais refrigerante, poderiam ter a absorção de cálcio prejudicada. Quanto a outros alimentos que prejudicam a absorção de cálcio (carnes e ovos - fontes de proteínas - e chá/café - fontes de cafeína), o presente estudo não demonstrou associação de sua ingestão com a de alimentos fontes de cálcio.

Ainda neste estudo, não se pôde demonstrar associação significativa entre a maior ingestão de leite pelos familiares e a maior ingestão por parte do aluno, embora se tenha obtido a informação de que os irmãos dos alunos avaliados ingerem mais leite do que seus pais.

Encontrou-se uma diferença importante, embora não significante, quanto à prática de atividade física entre os alunos que ingerem quantidades de cálcio conforme a *Food and Agriculture Organization* (FAO)/OMS^(^ ^13^ ^)^ e aqueles que não o fazem. Tal fato é extremamente importante, pois os alunos que costumam ingerir cálcio adequadamente também se beneficiam da maior absorção do mineral, uma vez que a prática de atividade física facilita sua absorção^(^ ^14^ ^)^.

Não se evidenciou diferença na ingestão de cálcio quanto aos sexos. Meninos e meninas equipararam-se quanto às quantidades de cálcio ingeridas, de modo similar aos estudos realizados em Osasco^(^ ^5^ ^)^ e Ouro Preto, MG^(^ ^9^ ^)^. Também não houve diferença na ingestão de cálcio entre alunos que frequentavam turnos diferentes (matutino e vespertino).

A osteopenia e a osteoporose são problemas comuns na idade adulta, mas podem ser prevenidas na adolescência por meio de hábitos alimentares adequados. Essa simples prática, além de proporcionar melhor qualidade de vida ao indivíduo, diminuiria os gastos com saúde por parte dos órgãos públicos^(^ ^15^ ^-^ ^18^ ^)^. É necessário, pois, um esforço entre os profissionais da saúde para estimular o aumento no consumo de alimentos ricos em cálcio entre os adolescentes, a fim de prevenir a osteoporose e suas consequências. Em Chapecó, após a devolução dos resultados desta pesquisa para as escolas, o cardápio escolar sofreu alterações para melhorar a oferta de produtos lácteos, tentando se adequar às necessidades diárias de cálcio na faixa etária estudada.

Conclui-se que a amostra, na sua maioria, não ingere níveis de cálcio adequados para a sua faixa etária e sexo, conforme as recomendações da FAO/OMS de 1300mg/dia^(^ ^1^ ^,^ ^13^ ^)^. 

As limitações deste estudo se encontram na aplicabilidade do questionário, pois se dependia do preenchimento completo e fidedigno pelos adolescentes. À semelhança de outros trabalhos citados, esta pesquisa demostra a baixa ingestão de cálcio na população de adolescentes no município de Chapecó, o que é um problema de Saúde Pública não apenas local.

 A partir deste estudo, torna-se necessário desenvolver um programa de incentivo à maior ingestão de cálcio na faixa avaliada. O fornecimento de informações e exemplos práticos aos jovens e a seus familiares/cuidadores sobre as fontes de alimentos ricos em cálcio, sua quantidade em cada alimento e os facilitadores e dificultadores da sua absorção, tornará mais fácil o entendimento acerca da importância da formação e manutenção da densidade mineral óssea na prevenção da osteopenia e da osteoporose.
